# Supplementary material for: Polyamine-Rich Diet Elevates Blood Spermine Levels and Inhibits Pro-Inflammatory Status: An Interventional Study
Source: Med Sci (Basel). 2021 Mar 29;9(2):22. doi: 10.3390/medsci9020022 (PMC8103277; doi:10.3390/medsci9020022)
Supplement: Supplementary file 1 [file medsci-09-00022-s001.pdf]

medical sciences

MDPI

Article

Polyamine-Rich Diet Elevates Blood Spermine Levels and Inhibits Pro-Inflammatory Status: An Interventional Study

Kuniyasu Soda [1,*], Takeshi Uemura [2], Hidenori Sanayama [1], Kazuei Igarashi [2,3] and Taro Fukui [1]

[1] Department Cardiovascular Institute for Medical Research, Saitama Medical Center, Jichi Medical University, 1-847, Amanuma, Saitama-city, Saitama 330-0834, Japan; sanayama@jichi.ac.jp (H.S.); d1423@jichi.ac.jp (T.F.)

[2] Amine Pharma Research Institute, Innovation Plaza at Chiba University, 1-8-15 Inohana, Chuo-ku, Chiba, 260-0856, Japan; uemura@amine-pharma.com (T.U.); iga16077@faculty.chiba-u.jp (K.I.)

[3] Graduate School of Pharmaceutical Sciences, Chiba University, 1-8-1 Inohana, Chuo-ku, Chiba 260-8675, Japan

* Correspondence: soda@jichi.ac.jp; Tel.: +81-48-647-2111

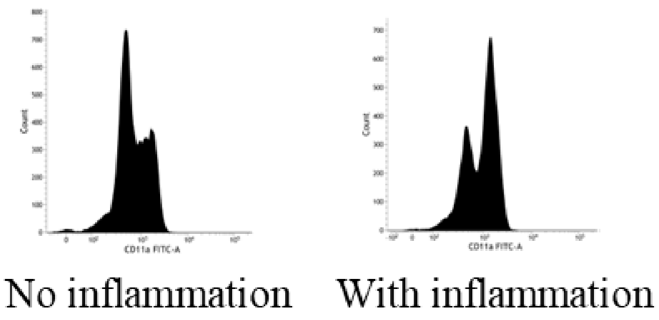

Figure S1. Histogram of CD11a expressions on PBMCs obtained in a normal (**left**) and febrile state (**right**) of the same volunteer.
